# Novel Insights into the Vasoprotective Role of Heme Oxygenase-1

**DOI:** 10.1155/2012/127910

**Published:** 2012-02-22

**Authors:** Emanuela Marcantoni, Luigia Di Francesco, Melania Dovizio, Annalisa Bruno, Paola Patrignani

**Affiliations:** ^1^Department of Medicine and Aging, School of Medicine, “G. d'Annunzio” University, 66100 Chieti, Italy; ^2^Center of Excellence on Aging (CeSI), School of Medicine, “G. d'Annunzio” University, 66100 Chieti, Italy; ^3^Department of Neuroscience and Imaging, School of Medicine, “G. d'Annunzio” University, 66100 Chieti, Italy

## Abstract

Cardiovascular risk factors contribute to enhanced oxidative stress which leads to endothelial dysfunction. These events trigger platelet activation and their interaction with leukocytes and endothelial cells, thus contributing to the induction of chronic inflammatory processes at the vascular wall and to the development of atherosclerotic lesions and atherothrombosis. In this scenario, endogenous antioxidant pathways are induced to restrain the development of vascular disease. In the present paper, we will discuss the role of heme oxygenase (HO)-1 which is an enzyme of the heme catabolism and cleaves heme to form biliverdin and carbon monoxide (CO). Biliverdin is reduced enzymatically to the potent antioxidant bilirubin. Recent evidence supports the involvement of HO-1 in the antioxidant and antiinflammatory effect of cyclooxygenase(COX)-2-dependent prostacyclin in the vasculature. Moreover, the role of HO-1 in estrogen vasoprotection is emerging. Finally, possible strategies to develop novel therapeutics against cardiovascular disease by targeting the induction of HO-1 will be discussed.

## 1. Introduction

For many years, atherosclerosis was considered an age-related process characterized by the passive accumulation of lipids in the vessel wall. However, the most recent lines of evidence have clearly shown that it is a complex process in which multiple pathogenic factors contribute to trigger and sustain vessel wall damage, leading to myocardial infarction, stroke, and sudden death [1]. In particular, there is an increasing appreciation of atherosclerosis as a dynamic and progressive disease starting with endothelial dysfunction which may trigger platelet activation and their interaction with leukocytes and endothelial cells. This process may contribute to the induction of chronic inflammation at the vascular wall [2].

Several lines of evidence suggest that oxidative stress may promote endothelial dysfunction through increased production of reactive oxygen species (ROS). Increased levels of diverse ROS are produced in the vessel wall and they individually or in combination may contribute to the pathogenesis of vascular disease. Thus, increased lipid peroxidation has been identified as a key mechanism for the development of atherosclerosis and inflammatory vascular damage. In fact, intracellular oxidative signals may induce the expression of a selective set of vascular inflammatory genes thus linking oxidative stress and inflammation in atherogenesis [3, 4].

Endothelial cells generate several protective mediators to regulate the functions of underlying vascular smooth muscle cells and circulating cells [5]. Among them, cyclooxygenase (COX)-2-dependent prostacyclin (PGI_2_) plays a central role [5]. COX-2 is among endothelial genes upregulated by steady laminar shear stress (LSS) [6], which characterizes “atherosclerotic lesion-protected areas” [7]. COX activity of the enzyme catalyzes the conversion of free arachidonic acid to prostaglandin (PG)G_2_, which is then converted to PGH_2_ through its peroxidase activity [8]. Endothelial cells may transform PGH_2_ to a different array of the prostanoids (i.e., PGD_2_, PGE_2_, and PGI_2_) along the vascular beds; however, robust evidence sustains that PGI_2_ is the dominant prostanoid produced in the macrocirculation [4, 9]. PGI_2_ exhibits properties of relevance to atheroprotection. In fact, it acts as a general restraint on endogenous stimuli to platelet activation, vascular proliferation and contraction, and cell adhesion [4]. It has been reported that PGI_2_ has antioxidant function before and in the early stage of atherogenesis through the induction of the antioxidant enzyme heme oxygenase (HO)-1 [10].

Recently, we provide evidence that COX-2-dependent PGI_2_ (induced by steady LSS) upregulates HO-1, which halts the proatherogenic cytokine, tumor necrosis factor (TNF)-*α*, in human endothelial cells [11]. Altogether, these data strongly support the key role of HO-1 pathway in the vasoprotective phenotype induced by PGI_2_.

In this paper we aim (i) to summarize the major features of the biology of HO-1 system by relating them to the role of this antioxidant enzyme in normal and pathological states, such as vascular inflammation and angiogenesis; (ii) to shed some light on the molecular mechanisms involved in the interplay between HO-1 system and the vasoprotective PGI_2_.

## 2. Biology of HO

 HO plays a central role in regulating the levels of intracellular heme by catalyzing the oxidative degradation of heme to liberate free iron, carbon monoxide (CO), and biliverdin in mammalian cells [20]. Biliverdin is metabolized to bilirubin by biliverdin reductase. Excess free heme catalyzes the formation of ROS, which leads to endothelial dysfunction as seen in numerous pathologic vascular conditions including systemic hypertension and diabetes, as well as in ischemia/reperfusion injury. The HO system, through its products, may cause different effects on the vascular system: (i) prevention of endothelial cell apoptosis; (ii) attenuation of the inflammatory response in the vessel wall; (iii) regulation of the vascular tone; (iv) participation in angiogenesis and vasculogenesis. Among all products of HO-1, bilirubin and biliverdin are the most potent endogenous scavengers of ROS [21] and CO exerts antiapoptotic and anti-inflammatory effects through the induction of soluble guanylyl cyclase. It suppresses the production of TNF-*α*, interleukin (IL)-1*β* and CCL4 chemokine (macrophage inflammatory protein-1*β*) and induces the synthesis of anti-inflammatory IL-10 [22]. Finally, free iron, despite participation in Fenton reaction that leads to formation of highly reactive hydroxyl radicals, activates Fe-ATPase. It is a transporter that removes intracellular iron as well as induces expression of ferritin heavy chains which sequester free iron and exert specific cytoprotective roles [23].

Three isoforms of HO have been described: an inducible isoform, HO-1, and two constitutively expressed isoforms, HO-2 and HO-3. HO-1 is a 32 kDa microsomal protein considered to be a protective, early stress-response agent that may have additional nonenzymatic activities related to its mitochondrial localization and nuclear translocation. The expression of HO-1 is generally very low in normal tissues, apart from liver and spleen, where it participates in the processing of senescent or damaged erythrocytes and in protection against oxidative damage caused by free porphyrins [24]. In all tissues, low basal expression of HO-1 can be upregulated by a wide variety of stimuli that cause oxidative stress, including its substrate heme, heavy metals, cytokines, ultraviolet rays, lipopolysaccharide, hydrogen peroxide, growth factors, nitric oxide (NO), and also CO [25]. HO-2, a 36-kDa protein which is constitutively expressed, is localized primarily in the brain, testis, and vascular endothelium [26, 27]. Recently it has been postulated a novel role for HO-2 in the regulation of the inflammatory and reparative response to injury, which is a cytoprotective mechanism typically associated with HO-1 induction. HO-2 may constitute an essential protective circuit responsible of a basal tone of anti-inflammatory signals critical to the execution of self-resolving inflammatory-reparative processes [28]. HO-3, a lastly cloned 33-kDa protein, which is a pseudogene derived from HO-2 transcript, has been found only in rats [29].

## 3. Regulation of HO-1 Gene Expression

There are different mechanisms involved in the modulation of HO-1 expression.

It has been reported that mitogen-activated protein kinases (MAPKs), phosphatidyl inositol 3-kinase/Akt, protein kinase (PK)A, PKC, and PKG [30], nuclear factor E2-related factor 2 (Nrf2), Bach1 (bric-a-brac, tramtrack, and broad complex and cap “n” collar homology 1), activator protein-1 (AP-1), nuclear factor-*κ*B (NF-*κ*B), cyclic adenosine monophosphate-responsive element-binding protein, and activating transcription factor 2 (ATF-2) [31] participate in HO-1 gene regulation. The transcription factor Nrf2 plays a central role in the transcriptional activation of HO-1 and many other genes encoding phase II drug-metabolizing enzymes in response to oxidative stress. Activation of Nrf2 is regulated by the cytosolic protein Keap1 that negatively modulates the nuclear translocation of Nrf2 and facilitates degradation of Nrf2 via the proteasome. Upon activation, Nrf2 enters the nucleus where it binds to the AU-rich elements (AREs) in the HO-1 promoter to trigger gene expression [17]. Nrf2 has been recently reported to regulate the induction of HO-1 in response to various forms of cellular stress, including hemodynamic, oxidative, and endoplasmic reticulum stress [32–34]. Moreover, fibroblasts and lung tissue from Nrf2-deficient animals express reduced levels of HO-1 [35, 36], further implicating Nrf2 in the induction of HO-1 [37].

Other transcription factors have been identified, such as the transcription factor Yin Yang** (**YY)1 that is a downstream effector of CO produced by HO-1 [38] and hypoxia-inducible factor (HIF)-1. It has been found that the increase in the transcription factor YY1 is involved in the inhibition of neointimal hyperplasia in vivo by HO-1 [38] and that the HIF-1 stabilization induces cardioprotection via HO-1 expression [39]. The results of Dawn and Bolli [39] show that HIF-1-mediated upregulation of HO-1 is beneficial to the ischemic myocardium.

In addition to the transcriptional regulation, it has been reported that a posttranslational mechanism may exist to attenuate HO-1 expression. It has been demonstrated that two miRNAs, miR-217 and miR-377, combine to attenuate HO-1 protein expression, resulting in a significant reduction in HO-1 enzyme activity. The knockdown of both miR-217 and miR-377 increases HO-1 protein expression, while the overexpression of the same miRNAs leads to attenuation of protein expression [40]. Recently, Lin et al. show that HO-1 is subjected to posttranslational regulation by the ubiquitin-proteasome system through an endoplasmic reticulum-associated degradation pathway [41]. Proteasome inhibition significantly decreased HO-1 protein degradation. Increased HO-1 expression by MG-132, a proteasome inhibitor, has been shown to protect astrocytes from heme-mediated oxidative injury [42].

## 4. Polymorphisms in HO-1 Gene

Three polymorphisms in the 5′ flanking region of the HO-1 gene have been described: a (GT)n dinucleotide length polymorphism [43] and two single-nucleotide polymorphisms (SNPs), G(-1135)A and T(-413)A [44]. Only two, the (GT)n repeat polymorphism and the T(-413)A SNP, have been reported to exert functional importance by influencing the level of HO-1 expression in different organ systems. Thus, they may enhance or suppress the susceptibility to various disease conditions, including the maintenance of pregnancy [44, 45] and various cardiovascular (CV) disease [44].

In view of the apparently beneficial effects of placental HO-1 expression for the pregnancy outcome, the relationship between idiopathic recurrent miscarriage and a (GT)n repeat microsatellite polymorphism of HO-1 gene has been investigated [45]. The results from this study firstly showed the association between the HO-1 (GT)n microsatellite polymorphism in the human HO-1 promoter regulatory region and women with idiopathic recurrent miscarriage in a relatively large Caucasian population, supporting the hypothesis that HO-1 polymorphisms among human population might contribute to some unexplained cases of pregnancy disorders, such as fetal growth retardation and preeclampsia [46].

HO-1 plays a critical role in protecting the CV system from the damaging effects of oxidative stress. The two functional polymorphisms of HO-1 gene have been associated with CV disease and have different frequency distributions based upon ethnicity [44]. In particular, a significant association between the AA genotype of a T(-413)A polymorphism and arterial hypertension in Japanese women, but not in men, was observed [47]. This polymorphism was suggested to be associated with a higher expression of HO-1 and the authors suggest that an interaction between estrogen-induced expression of NO synthase and HO-1-derived CO, which attenuates NO-induced vasodilation, may explain their findings [47]. However, the inconsistency between men and women raises some doubts on the reproducibility of these data. Moreover, the same authors demonstrated that the AA genotype of the T(-413)A polymorphism may reduce the incidence of ischemic heart disease, even if it may potentially increase the risk of hypertension [48].

(GT)n dinucleotide repeat in the HO-1 gene promoter shows a length polymorphism that modulates the level of gene transcription [43]. Compared with long (GT)n repeats, short (GT)n repeats in the human HO-1 gene promoter were shown to have higher transcriptional activity in response to oxidative stress [49]. It has been shown that length polymorphism in the HO-1 gene promoter is related to coronary artery disease susceptibility in Japanese people, but this association was found only in patients with hypercholesterolemia or diabetes mellitus or in smokers [50], thus suggesting that HO-1 may play an antiatherogenic role in Japanese patients with these coronary risk factors.

Moreover, (GT)n microsatellite polymorphism was reported to be associated with emphysema, restenosis after percutaneous transluminal angioplasty, and coronary artery disease [49, 51, 52]. However, in some studies the association between HO-1 polymorphisms and CV disease was not confirmed. A study based on a large number of 1807 patients showed that the (GT)n dinucleotide repeats length polymorphism located in the promoter region of the human HO-1 gene is not associated with the development of restenosis and major adverse clinical events following coronary stenting [53]. Similarly, Turpeinen et al. [54] showed that HO-1 gene polymorphisms have no significant role in outcome of kidney transplantation in the Finnish population. A recent prospective case-control study of more than 3000 participants showed that neither the (GT)n dinucleotide repeat nor the T(-413)A polymorphism in the HO-1 promoter is associated with angiographic coronary artery disease, myocardial infarction, or survival rate in Caucasians undergoing coronary angiography [55]. Thus, altogether these studies leave still open the debate about the functional relevance of both variants of polymorphisms in HO-1 promoter. It is not unusual that studies of genetic polymorphisms produce divergent results, especially if small numbers of cases and controls are examined; often positive associations seen in small studies have been disproven in subsequent larger studies. In conclusion, even if the regulation of HO-1 gene may be determined, at least in part, by genetics, neither the (GT)n dinucleotide repeat nor the T(-413)A polymorphism of the HO-1 gene can be considered reliable genetic markers for CV disease.

## 5. Role of HO-1 in Vascular Inflammation

HO-1 represses inflammation by removing the proinflammatory molecule heme and by generating CO and the bile pigments, biliverdin, and bilirubin. These HO-1 reaction products are capable of blocking innate and adaptive immune responses by modifying the activation, differentiation, maturation, and/or polarization of numerous cell types, including endothelial cells, monocytes/macrophages, dendritic cells, T lymphocytes, mast cells, and platelets. These cellular actions by CO and bile pigments result in diminished leukocyte recruitment and infiltration, and proinflammatory mediator production within atherosclerotic lesions [56].

The role of HO-1 in inflammation is demonstrated in HO-1 knockout mice, in which HO-1 deficiency leads to increased production of proinflammatory cytokines [57]. In patients subjected to bypass surgery, a higher activity of HO-1 resulted in a lower concentration of IL-6 [58]. HO-1 has been reported to reduce inflammatory cell rolling, adhesion, and migration from the vascular compartment, by downregulating the function and expression of adhesion molecules on the vessel wall [59, 60]. In contrast, inhibition of HO-1 increases adhesion molecule expression [61–63].

Preclinical and clinical evidence clearly suggests that the progression of atherosclerosis is associated with inflammation [64]. Different studies have been performed to understand whether HO-1 can be protective in the pathogenesis of this disease. Experimental evidence demonstrates that the induction of HO-1 in vascular cells suppresses oxidized low-density-lipoprotein (LDL)-induced monocyte transmigration and inhibits atherosclerotic lesion formation in LDL receptor (LDLR) knockout mice [65, 66]. Interestingly, the levels of bilirubin in the normal human population correlate inversely with the incidence of atherosclerotic events [67] and it has been shown that bilirubin attenuates vascular endothelial activation and dysfunction in vitro [12].

Recent interest has also focused on peroxisome proliferator-activated receptor *δ* (PPAR*δ*) ligands and induction of HO-1 expression. Ali et al. showed for the first time in vivo that PPAR*δ* ligands induce vascular endothelial HO-1 expression, thus supporting the hypothesis that PPAR*δ* represents an important potential target for the treatment of endothelial dysfunction and atherogenesis [13]. Finally, it has been shown that, in human monocytes, HO-1 activity is involved in attenuation of TNF-*α* production [68].

## 6. Cross-Talk between HO-1 and PGI_2_

PGI_2_ is considered a major prostanoid generated in the macrocirculation (both in endothelial cells and vascular smooth muscle cells) [5, 9], where it inhibits platelet activation, vascular smooth muscle cell contraction and proliferation, leukocyte-endothelial cell interactions [69], and cholesteryl ester hydrolase and induces thrombomodulin, an important inhibitor of blood coagulation [70, 71]. PGI_2_ acts mostly through I prostanoid receptor (IP), a rhodopsin-like class A, 7-transmembrane-spanning G-protein-coupled receptor (GPCR), which activates membrane-bound adenylyl cyclase and the subsequent formation of the second messenger cyclic adenosine monophosphate (cAMP) [14]. Recently, studies in animal experimental models have shown that COX-2-derived PGI_2_ confers atheroprotection in female mice lacking the LDLR (an animal model of atherosclerosis), through the induction of HO-1 [10]. However, the possible contribution of endothelial COX-1 to PGI_2_ biosynthesis and of endothelial COX-2 to the generation of other prostanoids, in particular PGE_2_ [72], has not been completely clarified. In fact, recent results suggest a cardioprotective role of PGE_2_ via E prostanoid receptors (EP)2 and EP4 [73, 74]; on the other hand, it is important to underline that PGE_2_, due its important role in inflammation, may enhance plaque burden and plaque destabilization in humans [75].

**Figure 1:**
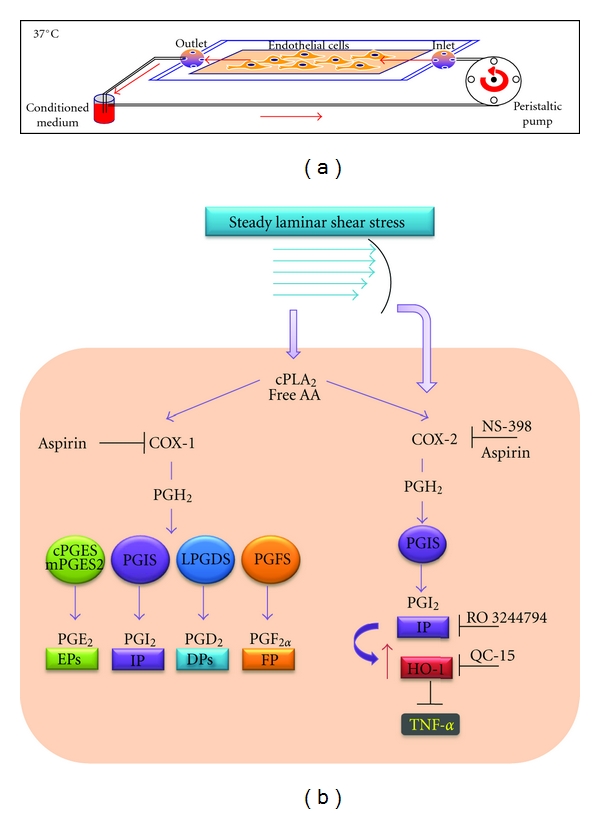
Exposure of endothelial cells to steady laminar shear stress (LSS). (a) HUVECs (0.8 to 1 × 10^6^ cells per glass slide) are shear stressed using a parallel plate flow chamber connected to a constant pressure drop flow loop, maintained at 37°C and gassed continuously with a humidified mixture of 5% CO_2_ in air. Endothelial monolayers are continuously perfused in a closed circuit at an estimated shear stress of 10 dyn/cm^2^ (flow rate of 2.53 mL/min; shear rate of 1400 sec^−1^) with 7 mL of perfusion DMEM-medium199 (50% vol/vol), supplemented with 5% fetal calf serum, 1% glutamine, and antibiotics for 6 hours [11]. (b) In HUVEC, steady LSS activates cPLA_2_, thus releasing free arachidonic acid (AA) from cell membrane phospholipids, the substrate of cyclooxygenase isoenzymes (COX-1 and COX-2). In addition, LSS upregulates COX-2 expression in HUVEC, without affecting the expression of COX-1 and downstream synthases (such as cPGES, mPGES2, PGIS, LPGDS, PGFS) [11]. Both COX-1 and COX-2 participate in the biosynthesis of PGE_2_, PGI_2_, PGD_2_, and PGF_2*α*_ as suggested by the finding that aspirin (a nonselective COX inhibitor) affects the levels of all these prostanoids. Differently, the selective COX-2 inhibitor (NS-398) affected only PGI_2_ in HUVECs exposed to LSS which overexpressed COX-2. COX-2-dependent PGI_2_, induced by LSS, through the interaction with a specific receptor (IP), causes the induction of HO-1. It constrains TNF-*α* biosynthesis in HUVECs under this experimental condition. In fact, LSS-dependent reduction of TNF-*α* generation is completely countered by the selective COX-2 inhibitor NS-398, the nonselective COX inhibitor aspirin, or the specific PGI_2_ receptor (IP) antagonist RO3244794 [11, 12]. Finally, by the use of the novel imidazole-based HO-1 inhibitor QC15 [13], it has been shown that HO-1 induction in response to COX-dependent PGI_2_ plays a role in LSS-dependent reduction of TNF-*α* biosynthesis [11].

Thus, recently, we performed a study in human umbilical vein endothelial cells (HUVECs) exposed a physiological fluid mechanical stimulus in vitro [11] (Figure 1(a)) with the aims to (i) distinguish between the vasoprotective function of COX-2 and COX-1 and (ii) evaluate the contribution of different prostanoids to endothelial vasoprotection. In this study, we showed that in HUVECs exposed to uniform LSS of 10 dyn/cm^2^ (characteristically associated with lesion-protected areas), COX-2, but not COX-1 and downstream synthases, was significantly induced, and this translated into enhanced biosynthesis not only of PGI_2_, but also of other prostanoids, such as PGE_2_ and PGD_2_ (Figure 1(b)). Pharmacological studies, using a selective COX-2 inhibitor (NS-398) and a nonselective COX inhibitor (aspirin), showed that both COX-2 and COX-1 contributed to PGI_2_ generation while only COX-1 contributed to PGE_2_ and PGD_2_. In the same study, we found that steady LSS reduces the synthesis and release of TNF-*α* (a known mediator of endothelial dysfunction and atherogenesis) [76, 77] from endothelial cells. Interestingly, we found that LSS-dependent reduction of TNF-*α* generation was completely countered by NS-398, aspirin, or the specific PGI_2_ receptor (IP) antagonist RO3244794 [78] (Figure 1(b)). Altogether, these results support the role of COX-2-dependent PGI_2_ in LSS-dependent reduction of endothelial TNF-*α* generation. Since LSS induced the expression of HO-1 and this effect was inhibited by NS-398, aspirin, or the IP antagonist, we hypothesized that the induction of HO-1, as a consequence of COX-2-dependent PGI_2_ generation, is involved in LSS-dependent reduction of endothelial TNF-*α* biosynthesis. This hypothesis was confirmed by the use of the novel imidazole-based HO-1 inhibitor QC15 [79]. In fact, we showed that the inhibition of HO-1 activity was associated with a complete abrogation of LSS-dependent inhibition of TNF-*α* biosynthesis (Figure 1(b)). Altogether these results support the contribution of LSS-induced PGI_2_ in the anti-inflammatory effect of HO-1 in endothelial cells. This seems to be a novel protective action of endothelial PGI_2_ which may work in physiological conditions [11].

**Figure 2:**
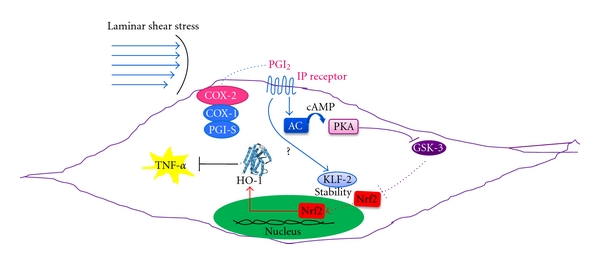
Postulated molecular mechanism involved in the induction of HO-1 by COX-2-dependent PGI_2_, in endothelial cells exposed to steady laminar shear stress (LSS). In endothelial cells exposed to uniform LSS (characteristically associated with atherosclerotic lesion-protected areas), COX-2 is overexpressed [11]. PGI_2_, mainly produced by the combined activity of COX-2 and PGI_2_-synthase (PGIS), interacts with its specific receptor, IP [14]. This interaction may lead to the activation of adenylate cyclase (AC), causing an increase of intracellular levels of cyclic AMP (cAMP) and subsequently to the activation of protein kinase A (PKA) [14]. PKA may phosphorylate glycogen synthase kinase(GSK)-3 [15], causing its inactivation and the loss of the capacity to phosphorylate nuclear factor E2-related factor 2 (Nrf2) [16]. Reduced phosphorylation of Nrf2 causes its stabilization and translocation into the nucleus, where it promotes the transcription of antioxidant and phase II genes, including HO-1 [11, 17]. In addition to Nrf2, Kruppel-like factor(KLF)-2 is increased in endothelial cells exposed to LSS [18]. KLF2 enhances antioxidant activity of Nrf2 by increasing its nuclear localization and activation [19]. The synergistic activity of these two transcription factors forms a major contribution to the shear-stress-elicited transcriptome in endothelial cells. The overexpression of HO-1 in endothelial cells by LSS exerts an anti-inflammatory action through its capacity to inhibit the biosynthesis and release of TNF-*α* [11].

Further specific studies have to be performed to clarify the molecular pathways involved in the regulation of the vasoprotective gene HO-1 by COX-2-dependent PGI_2_ in endothelial cells. We proposed that IP receptor signalling, through the activation of PKA, may induce the phosphorylation of glycogen synthase kinase (GSK)-3 [15], thus causing its inactivation and the loss of the capacity to phosphorylate Nrf2 [16]. This might translate into the stabilization of Nrf2 and its translocation into the nucleus, where it promotes the transcription of antioxidant and phase II genes, including HO-1 [11] (Figure 2). Furthermore, it has been shown that the Kruppel-like factor(KLF)-2 is increased in endothelial cells exposed to LSS [18]. This transcription factor may enhance antioxidant activity of Nrf2 by increasing its nuclear localization and activation [19]. The synergistic activity of the 2 transcription factors (Nrf2 and KLF-2) represents the major contribution to the shear-stress-elicited transcriptome in endothelial cells (Figure 2). Altogether our study provides evidence that COX-2-dependent PGI_2_ (induced by steady LSS) upregulates HO-1 which halts TNF-*α* generation in human endothelial cells [11]. This vasoprotective effect is abrogated by COX inhibitors, thus suggesting that inhibition of COX-2-dependent PGI_2_ might contribute to acceleration of atherogenesis in patients taking traditional (t) nonsteroidal anti-inflammatory drugs (NSAIDs) and NSAIDs selective for COX-2 (coxibs).

## 7. Role of HO-1 in Angiogenesis

Angiogenesis involves the formation of new blood vessels and is critical for fundamental events such as development and repair after injury [80]. Recently, it has been shown that HO-1 and its gaseous product CO have potent proangiogenic properties in addition to well-recognized anti-inflammatory, antioxidant, and antiapoptotic effects [80]. Angiogenic factors, such as vascular endothelial growth factor (VEGF) and stromal cell-derived factor-1 (SDF-1), mediate their proangiogenic effects through induction of HO-1, making it an attractive target for therapeutic intervention [80]. It has been reported that the role of HO-1 in angiogenesis regulation could be “good” or “bad.” The role of HO-1 in favoring angiogenesis responses is crucial for proper placental vascularization, wound healing, and neovascularization of ischemic heart. However, it may have detrimental outcomes in diseases where new blood vessel formation is undesirable, such as in tumor neovascularization [80].

Zhao and collaborators recently showed that a partial deficiency of maternal HO-1 resulted in the malformation of fetomaternal interface, alteration of the placental vasculature, insufficiency of spiral artery remodeling, and alteration of uterine natural killer cell differentiation and maturation [46]. These changes were independent of the fetal genotype, but relied on the maternal HO-1 level, which determined the balance of expression levels of pro- and antiangiogenic factors in the deciduas region [46]. According to these results a reduction in HO-1 placental expression was associated with recurrent miscarriages, spontaneous abortions, and preeclampsia [81]. These findings are in agreement with the results that HO-1 polymorphisms (as described above) are associated with idiopathic recurrent miscarriage in a relatively large Caucasian population of women [45].

The replacement of damaged capillaries and reestablishment of the normal oxygen amounts to a wound are accomplished by neovascularization. Wound-healing process includes a coagulation phase (characterized by endothelial dysfunction and platelet activation), an early extracellular matrix deposition, the release of factors by platelets, an inflammatory phase, and the resulting granulation, which are all events that rely on angiogenesis [82]. Growth factors including VEGF, chemokines like SDF-1, and hypoxia-inducible factors (HIFs) also coordinate the multifaceted events involved in wound healing [83, 84]. Interestingly, compared with wild-type littermate mice, HO-1-deficient mice exhibit impaired wound healing due, in part, to reduced recruitment of endothelial progenitor cells (EPCs) and capillary formation at the site of injury [85]. In addition, the induction of HO-1 in wounded skin was relatively weak and delayed in diabetic mice, in which also angiogenesis and wound closure were impaired. In such animals, local delivery of HO-1 transgene, using adenoviral vectors, accelerated the wound healing and increased the vascularization [86].

It has been recognized that HO-1 has a protective effect in ischemic myocardium by the increasing of expression of angiogenic growth factors in the infarcted tissue [87]. VEGF is a strong therapeutic reagent by inducing angiogenesis in ischemic myocardium [88], and it can mediate the ischemia-induced mobilization of EPCs from bone marrow [89]. Lin et al. showed that HO-1 gene transfer after myocardial infarction provides protection at least in part by promoting angiogenesis through inducing angiogenic growth factors [90]. In addition, preclinical and clinical studies have demonstrated that mesenchymal stem cells (MSCs) transplantation can attenuate ventricular remodeling and augment cardiac function when implanted into the infarcted myocardium. In HO-1-transfected MSCs-treated hearts, the myocardial apoptosis was marked with significantly reduced fibrotic area and the cardiac function and remodeling were also significantly improved [87].

It is important to point out that in addition to the numerous lines of evidence supporting the positive role of HO-1 in angiogenesis regulation, several authors reported of the negative effects of this enzyme in tumor angiogenesis. In particular, it has been shown that several human tumors, including renal cell and prostate cancer, express high levels of HO-1 [91, 92]. HO-1 may promote tumor cell survival [93], hindering the effectiveness of anticancer therapies [94]. In contrast, inhibition of HO-1 has been shown to enhance tumor regression in animal models [95], suggesting that the HO-1 pathway may be a therapeutic target in carcinogenesis [80].

However, in prostatic cancer cells (PC3), HO-1 seems to be antiangiogenic. In fact, Ferrando et al. [96] identified a set of inflammatory and proangiogenic genes downregulated in response to HO-1 overexpression, in particular VEGFA, VEGFC, HIF1*α*, and *α*5*β*1 integrin. An in vivo angiogenic assay showed that intradermal inoculation of PC3 cells stably transfected with HO-1 (PC3HO-1) generated tumors less vascularized than controls, with decreased microvessel density and reduced CD34 and MMP9 positive staining. Interestingly, longer-term grown PC3HO-1 xenografts displayed reduced neovascularization with the subsequent downregulation of VEGFR2 expression. Additionally, HO-1 repressed NF-*κ*B-mediated transcription, which strongly suggests that HO-1 may regulate angiogenesis through this pathway. Taken together, these data support a key role of HO-1 as a modulator of the angiogenic switch in prostate carcinogenesis ascertaining it as a logical target for intervention therapy [96].

## 8. Interplay between HO-1 and Estrogen

Estrogen has both rapid and longer-term direct effects on CV tissues mediated by the two estrogen receptors, ER-*α* and ER-*β* [97]. Estradiol promotes endothelial cell growth, protects endothelial cells against damage by oxidants and cholesterol, and induces the generation of endothelial-derived vasodilators, such as NO and prostanoids [98]. In fact, premenopausal women are less susceptible to myocardial infarction and stroke than are males of the same age group, an advantage that is lost after menopause [99]. Several animal studies and some small clinical trials support a cardioprotective action of estrogens [100, 101]. E_2_ retards atherogenesis in animal models [102] and improves endothelial disfunction in hyperlipidemic women [103].

Recently, Egan et al. [10] found that deletion of PGI_2_ receptor (IP) removes the atheroprotective effect of estrogen in ovariectomized female mice. The atheroprotective role of estrogen, in this setting, seems to be mediated by the induction of PGI_2_ biosynthesis. PGI_2_ activates its plasma membrane receptor IP which causes the induction of the antioxidant HO-1 in the vasculature. In fact, in vitro experiments, in mouse aortic smooth muscle cells (MASMCs), showed that estrogen acts on ER-*α* to upregulate the production of atheroprotective PGI_2_ through the induction of COX-2 [10]. MASMCs lacking the PGI_2_ receptor (IP/KO) showed an increased oxidative stress suggesting that IP modulates oxidant stress under basal conditions. In addition, cicaprost, an IP agonist, increased HO-1 protein expression in wild-type MASMCs but not in IP/KO MASMCs. The involvement of IP signalling in the induction of vascular HO-1 was shown also in vivo. Thus, IP deletion decreased aortic HO-1 protein expression in female mice lacking both the IP and the LDL scavenger receptor (LDLR) (IP/LDLR DKO) [10]. These data showed that the atheroprotective role of estrogen is mediated by enhanced generation of COX-2-dependent PGI_2_ and suggest that chronic treatment of patients with NSAIDs selective for COX-2 (coxibs) or tNSAIDs could undermine the estrogen-mediated protection from CV disease in premenopausal females. A study was performed to estimate the interaction, in a general population setting (using information from the UK's General Practice Research Database), between tNSAIDs and hormone therapy on the occurrence of acute myocardial infarction and death from coronary heart disease [101]. The researchers found that current use of hormone replacement therapy was associated with a lower risk of heart attack than nonuse. However, when looking at women who used tNSAIDs at the same time as hormone replacement therapy, the researchers found no suggestion of a reduction in risk of heart attack. These findings suggest that hormone therapy and NSAIDs might interact, with NSAIDs acting against a role for hormone replacement therapy in preventing heart attacks. This pharmacodynamic interaction might play a role, at least in part, in the uncertain results regarding the effect of postmenopausal hormone therapy on heart disease in women [104].

## 9. Cross-Talk between HO-1 and Cytochrome P-450-Derived Epoxyeicosatrienoic Acids

A molecular crosstalk between the cytochrome P-450-derived epoxyeicosatrienoic acids (EETs) and HO-1 gene expression was studied by Sacerdoti and coworkers [105, 106]. EETs induce HO-1 expression and signalling cascade [107], including activation of AMP-activated kinase (AMPK) and pAKT, thus reducing adiposity and insulin resistance in animal model of obesity and diabetes. In addition, EETs decrease MSC-derived adipocyte stem cell differentiation by the upregulation of HO-1-adiponectin-AKT signalling, suggesting that EET agonist may have potential therapeutic role in the treatment of dyslipidemia, diabetes, and the metabolic syndrome [108]. The potential action of EETs as intracellular lipid signalling modulators of adipogenesis was further supported by the recent finding that the treatment with EET agonists inhibits adipogenesis and decreases the levels of inflammatory cytokines. Interestingly, these effects are associated with the increase of HO-1 expression, which occurs through the inhibition of a negative regulator of HO-1 expression, Bach-1 [109].

## 10. Conclusions and Perspectives

Vascular health depends on a delicate balance in the vascular wall of prooxidative and antioxidant cellular mechanisms [10, 11]. Several lines of evidence have shown that HO-1 plays a central role in the vasoprotection effects of PGI_2_. COX-2-dependent PGI_2_ (induced by steady LSS) upregulates HO-1 which halts TNF-*α* generation in human endothelial cells [11]. Thus, clinical conditions associated with reduced generation of vascular PGI_2_ or the inhibition of COX-2-dependent PGI_2_ by coxibs and tNSAIDs may cause CV hazard [5], at least in part, through downregulation of HO-1 expression. In fact, HO system could attenuate/block the progression of vascular diseases via its antioxidant, anti-inflammatory, and antiproliferative effects.

Due to several beneficial effects of HO-1 for the CV system, it has emerged as a promising therapeutic target in the treatment of vascular disease. Pharmacological induction or gene transfer of HO-1 ameliorates vascular dysfunction in animal models of atherosclerosis, postangioplasty restenosis, vein graft stenosis, thrombosis, myocardial infarction, and hypertension, while inhibition of HO-1 activity or gene deletion exacerbates these disorders [110].

Gene therapy and gene transfer, including site- and organ-specific targeted gene transfer have become powerful tools for studying the potential role of HO-1 in the treatment of CV diseases. HO-1 induction by pharmacological agents or the in vitro gene transfer of human HO-1 into endothelial cells increases cell cycle progression and attenuates angiotensin II, TNF-*α*, and heme-mediated DNA damage. In addition, administration of human HO-1 to rats in advance of schemia/reperfusion injury considerably reduces tissue damage [111]. On the other hand, it should be point out that overexpression of human HO-1 may lead to some possible side effects. In particular, it may accelerate tumor growth, stimulates early stages of angiogenesis [80], increases the occurrence of metastasis and resistance to chemotherapy and photodynamic therapy [112].

Currently, gene therapy with the use of antioxidant genes, such as HO-1, is emerging as a promising approach for selecting CV pathologies, in particular for patient groups not suitable for conventional therapies [113]. However, in this area a further improvement of gene transfer vectors and transfer protocols to more efficiently transduce different cell types of the CV system is still required and diagnostic means for better identification of patients most likely to benefit from gene therapy interventions are lacking.
